# Preparation of Biomass-Based Iron-Containing Microspheres from Rice Straw: Enhancing the Water Absorption Performance of Slow-Release Fertilizer

**DOI:** 10.3390/gels12080700

**Published:** 2026-08-05

**Authors:** Chonghao Zhu, Tianhao Fang, Peiyao Na, Huiqing Li, Chenghai Liu, Xianzhe Zheng, Guoxiang Zheng, Shengming Zhang

**Affiliations:** 1 College of Agricultural Equipment and Energy Engineering, Northeast Agricultural University, Harbin 150030, China; 2 Key Laboratory of Pig-Breeding Facilities Engineering, Ministry of Agriculture and Rural Affairs, Harbin 150030, China; 3 Heilongjiang Provincial Engineering Research Center for Mechanization and Materialization of Major Crops Production, Harbin 150030, China; 4 Heilongjiang Provincial Key Laboratory of Technology and Equipment for the Utilisation of Agricultural Renewable Resources in Cold Region, Northeast Agricultural University, Harbin 150030, China

**Keywords:** rice straw, iron, microspheres, water absorption, slow-release

## Abstract

Reintroducing crop straw into soil boosts organic matter, but natural crop straw usually shows low nutrient content. To enhance the water absorption rate of a certain thin sheet-shaped iron-containing material, a technical approach combining ball milling, the Mannich reaction, Schiff’s base cross-linking, emulsion separation, and solid–liquid adsorption was attempted to prepare iron-containing microspheres using sodium alginate and rice straw as raw materials. This approach increased the content of the trace nutrient iron and improved the water absorption rate. The results indicated that the iron-containing microspheres exhibit a diameter ranging from approximately 15 μm, an iron content of 10.44%, and a water absorption rate of 501.76%. The release rates of iron and nitrogen in soil and water within the first day were all below 15%, while the release rates after 30 days in soil were 61.8% for iron and 44.32% for nitrogen, and the corresponding rates in water were 73.1% for iron and 63.88% for nitrogen. The water absorption capacity of the iron-containing microspheres showed a trend associated with particle size and pore structure parameters. The development of iron-containing microspheres has expanded the preparation technology for semi-interpenetrating structure type sustained-release materials, and holds the potential for further development into slow-release fertilizers.

## 1. Introduction

The incorporation of crop straw into agricultural soils is one of the principal measures for substituting commercial fertilizers with organic fertilizers, which is beneficial for the optimization of soil structure [1]. However, the nutrient content in crop straw is significantly lower than that of commercial fertilizers. Moreover, directly returning a large amount of straw to the field might result in an increase in diseases and pests and a reduction in crop production [2]. The straw and its main components are used to produce coating materials that encapsulate nutrients. Through physical barriers or hydrophobic effects, the release of nutrients can be delayed [3,4]. The conversion of straw into carbon-rich porous materials through hydrothermal carbonization technology has also been applied in soil improvement and environmental management [5]. However, the aforementioned method requires a complete disruption of the lignocellulose structure of the biomass, which poses a problem of complex production processes.

The cellulose, lignin, and hemicellulose in the straw are interconnected through chemical bonds and physical interactions, forming a three-dimensional polymer [6]. Some substances with interpenetrating and semi-interpenetrating structures have been proven to be able to serve as carriers for achieving the sustained release of nutrients [7,8]. Iron is an essential micronutrient involved in chlorophyll formation, but excessive iron can disrupt plant metabolism [9]. Doping gel materials with biomass to form composite material carriers helps control the slow release of nutrients. Pan et al. [10] synthesized a hydrogel “CPAUH” with semi-interpenetrating structure characteristics using carboxylated cellulose, acrylamide, and potassium acrylate, achieving the slow release of urea and humic acid over a period of 15 days. In our previous research, iron-loaded oxidized alginate was grafted onto the lignin framework of rice straw, resulting in a thin sheet-shaped material that achieved a controlled slow release of iron within 28 days [11]. However, this material has the drawback of having insufficient water absorption capacity.

Enhancing the water absorption of fertilizer materials is beneficial for improving local water availability around fertilizer particles and can contribute to crop growth [12]. In particular, the root systems of plants tend to grow towards areas with a more abundant water supply [13]. The organic acids secreted by the roots can participate in the reduction in trivalent iron, and this effect helps to increase the effective utilization rate of iron [14]. Sodium alginate is rich in carboxyl and hydroxyl groups, possessing unique cross-linking properties and excellent swelling performance [15]. It has been utilized to prepare porous films, hydrogels, microspheres, and other materials, playing a significant role in the fields of biomedicine and chemical industry [16,17]. In particular, through functional modification, the number of active sites of sodium alginate can be increased, thereby further enhancing the service life and performance of the material [18]. Moreover, the physical behavior and properties of polymers are influenced by the dimensional scale and interfacial interactions, and the behavior of materials at the mesoscopic, microscopic, and nanoscopic levels differs from that in the macro or super-scale domains [19].

Based on the above considerations, to address the issue of poor water absorption of the thin sheet-shaped iron-containing materials, this study proposes to innovatively prepare iron-containing microspheres. It is expected to control the water absorption performance and nutrient release of the iron-containing microspheres by adjusting their particle size, which is derived from the oxidized sodium alginate gel and rice straw. Based on the previous research that involved in situ amidation of rice straw through the Mannich reaction, Schiff’s base cross-linking reaction, and solid–liquid adsorption principle to prepare thin sheet-shaped iron-containing materials, this study systematically investigated the method of constructing microspheres through the emulsion polymerization process. Response surface methodology was used to optimize two primary material parameters, namely iron content and water absorption capacity, while the release behavior and pot-scale seedling growth response were further evaluated using the optimized formulation. The preparation of iron-containing microspheres can expand the preparation technology of semi-interpenetrating sustained-release materials using oxidized sodium alginate gel and straw as raw material sources, and provide an initial understanding for the development of iron-containing microspheres into sustained-release fertilizers.

## 2. Results and Discussion

### 2.1. Optimal Preparation Conditions of the Iron-Containing Microspheres

**Figure 1:**
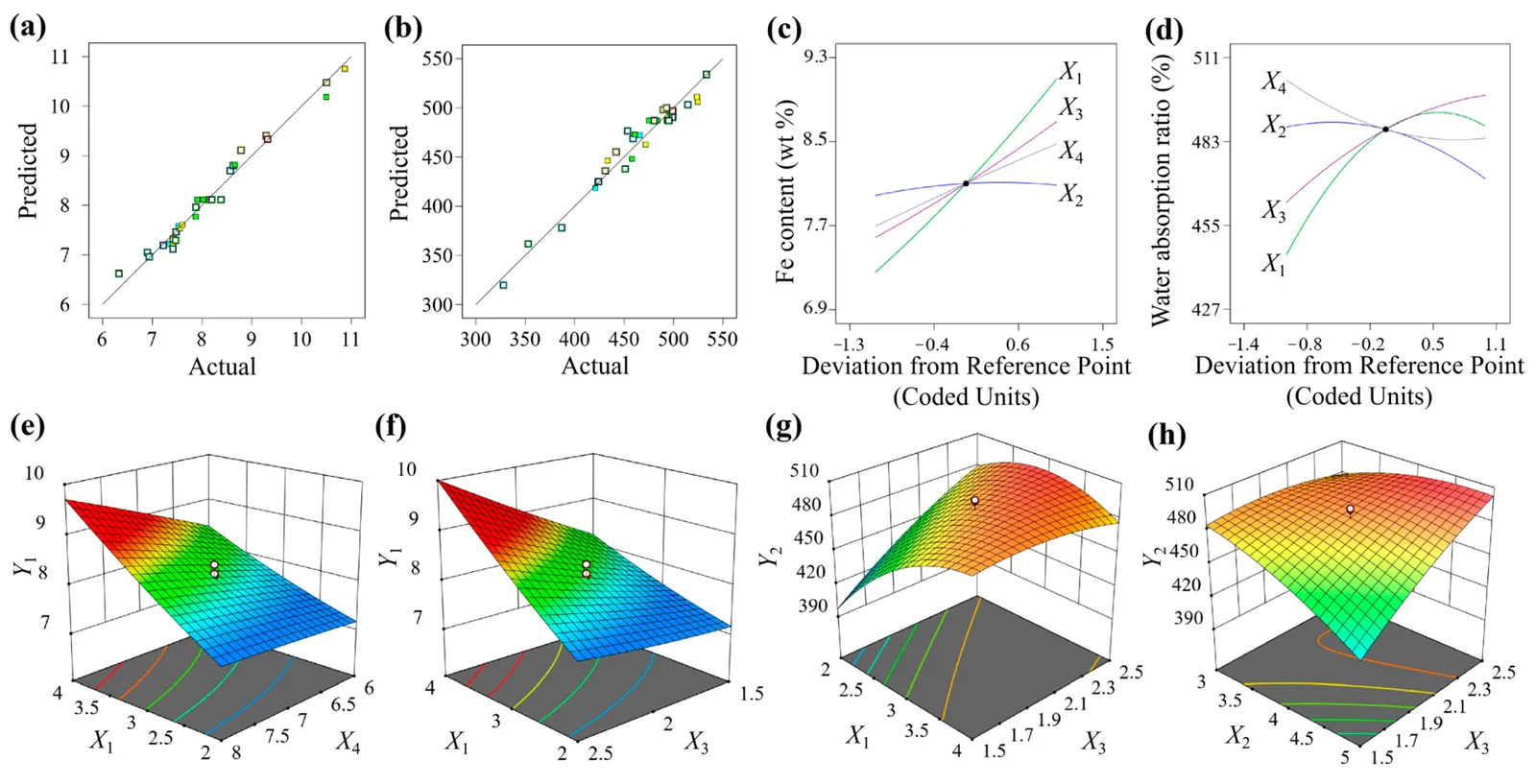
Relationship between predicted and actual responses of iron content (**a**) and water absorption rate (**b**); the perturbation charts of iron content (**c**) and water absorption rate (**d**); response-surface plots showing the effects of X_1_X_4_ (**e**) and X_1_X_3_ (**f**) on iron content; response-surface plots showing the effects of X_1_X_3_ (**g**) and X_2_X_3_ (**h**) on water absorption rate.

Iron content and water absorption capacity were selected as the response variables because they correspond to the two main design targets of the microspheres: iron-loading capacity and water-absorbing performance. The impact of preparation conditions on the iron content and water absorption rate of iron-containing microspheres, as well as the comparison between predicted and actual values for these parameters, is illustrated in Figure 1. The rice straw powder was ground for 12 h and utilized as the raw material, which was recorded as RS. The corresponding iron content, water absorption rate, and analysis of variance (ANOVA) results are presented in Tables S1 and S2. ANOVA results from the response surface quadratic model indicated that lack-of-fit values (*p*-value of iron content = 0.2035 > 0.05, *p*-value of water absorption rate = 0.0863 > 0.05) were not significant; meanwhile, the model itself was highly significant (*p*-value of iron content < 0.0001, *p*-value of water absorption rate < 0.0001), indicating that the established regression model possessed reliability and excellent characterization ability. The regression coefficients were subjected to significance testing, with non-significant terms being excluded at the α = 0.05 level. The optimized regression models for the iron content and water absorption rate of iron-containing microspheres were determined as follows:

(1) $$Y_{1}=+8.11+0.89X_{1}+0.54X_{3}+0.38X_{4}+0.37X_{1}X_{3}+0.27X_{1}X_{4}$$

(2) $$Y_{2}=+487.4+21.63X_{1}-8.68X_{2}+17.99X_{3}-9.78X_{4}+18.02X_{1}X_{2}-23.01X_{1}X_{3}+15.69X_{1}X_{4}+20.63X_{2}X_{3}$$

where *Y*_1_ represents the iron content of iron-containing microspheres expressed as wt%; *Y*_2_ represents the water absorption rate of iron-containing microspheres; *X*_1_, *X*_2_, *X*_3_, and *X*_4_ correspond to the coding in Table 1.

The determination coefficients of the regression models for iron content and water absorption rate were 0.9778 and 0.9555, respectively. The high determination coefficient indicates that the changes in iron content and water absorption rate of iron-containing microspheres were primarily influenced by independent variables. In Figure 1a,b, the predicted data closely matched the actual data, which indicated that the two regression models can effectively predict the iron content and water absorption rate in iron-containing microspheres. The ANOVA results indicated that among the four independent variables, the mass ratio of oxidized sodium alginate to aminated rice straw had the strongest influence on iron content compared to the other variables. For water absorption rate, X_1_ also showed the greatest influence, followed by the volume ratio of oil phase to aqueous phase (X_3_), whereas X_2_ and X_4_ showed comparatively weaker effects. Furthermore, in Figure 1c,d, the perturbation results for the four independent variables showed that under the conditions of X_1_, X_2_, X_3_, and X_4_, the difference between the highest and lowest iron content was 1.8, 0.1, 1.1, and 0.8, respectively. Similarly, the difference between the highest and lowest water absorption rates was 38, 29, 35, and 20, respectively. Thus, it can be concluded that the independent variable X_1_ was the most significant factor in affecting the iron content and water absorption rate of iron-containing microspheres.

In Figure 1e,f, the effect of interaction terms X_1_X_4_ and X_1_X_3_ on iron content showed that, with X_2_ held constant, the iron content of microsphere samples increases as the mass ratio of OSA to ARS, the volume ratio of oil phase to aqueous phase, and the weight concentration of Fe(NO_3_)_3_ solution increase. In Figure 1g,h, the impact of interaction terms X_1_X_3_ and X_2_X_3_ on water absorption rate showed that the water absorption rate of microspheres samples was primarily influenced by the mass ratio of OSA to ARS, the ratio of total mass of OSA and ARS to the mass of deionized water, and the volume ratio of oil phase to aqueous phase, with minimal correlation to the weight concentration of Fe(NO_3_)_3_ solution. This observation aligns with the ANOVA results. Based on calculations from the quadratic regression model, it is projected that the theoretical maximum iron content in iron-containing microspheres is 10.75%, while achieving a water absorption rate of 511%. The predicted reaction conditions are as follows: the mass ratio of OSA to ARS is 4:1, the ratio of the total mass of OSA and ARS to the mass of deionized water is 1:5, the volume ratio of oil phase to aqueous phase is 2.5:1, and the weight concentration of Fe(NO_3_)_3_ solution is 8%. As shown in Table 2, the results of the confirmatory experiment showed that under these reaction conditions, the iron content and water absorption rate of the iron-containing microspheres were 10.44% and 501.76%, respectively. The predicted values were within the 95% confidence intervals of the experimental results, and the relative errors between the predicted and experimental mean values were 2.97% for iron content and 1.84% for water absorption rate, indicating good agreement between the model prediction and independent-batch validation.

### 2.2. Characterization of Iron-Containing Microspheres

**Figure 2:**
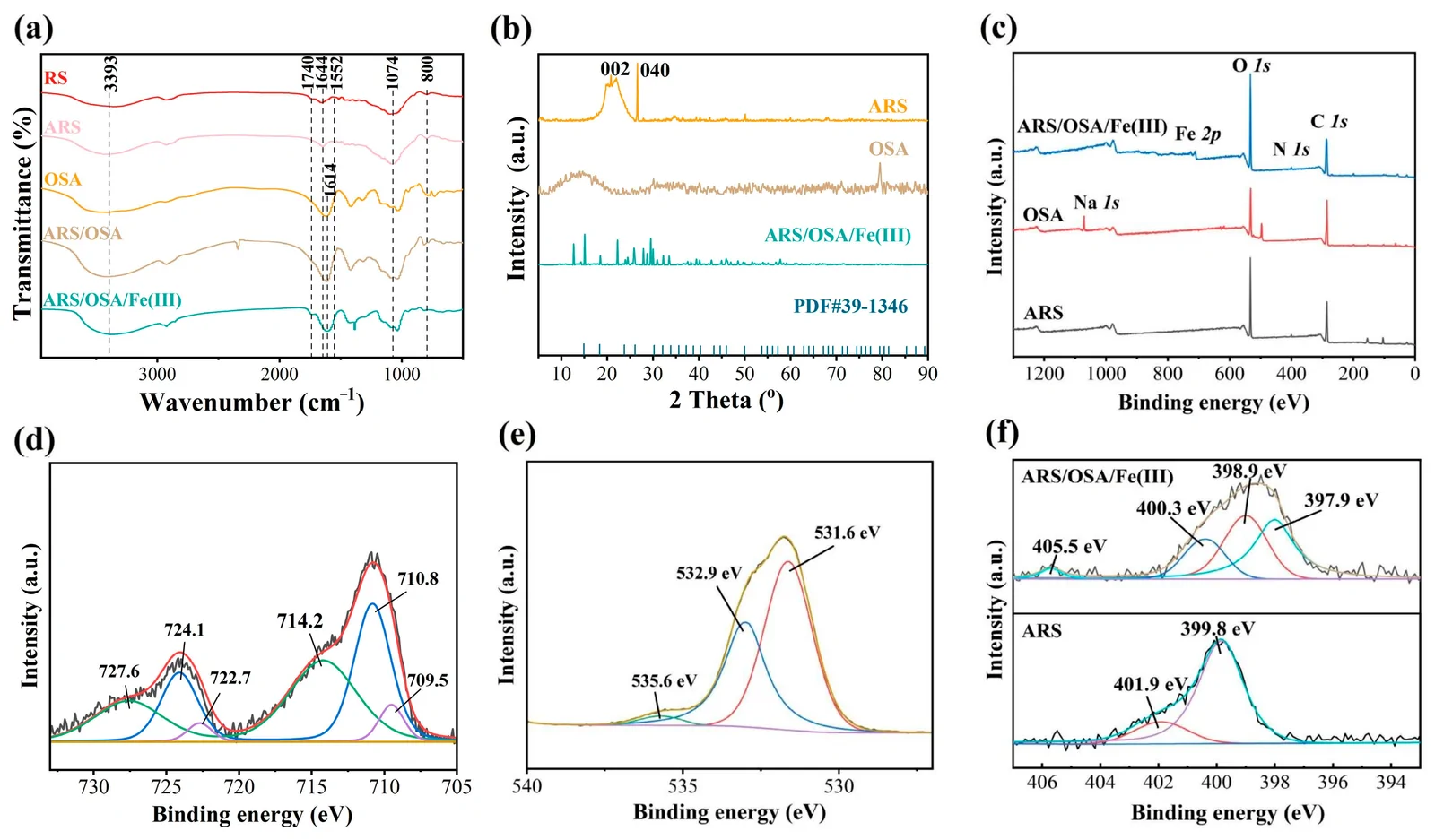
Characterization of iron-containing microspheres and other samples: FT-IR spectra (**a**), XRD patterns (**b**), X-ray photoelectron spectroscopy (XPS) survey spectra (**c**), high-resolution Fe 2p spectrum of iron-containing microspheres (**d**), high-resolution O 1s spectrum of OSA (**e**), high-resolution N 1s spectra of ARS and iron-containing microspheres (**f**).

The Fourier transform infrared (FT-IR) spectra of iron-containing microspheres and other samples are presented in Figure 2a. Compared with RS, the infrared spectrum of ARS exhibited pronounced changes in the amine-associated region. The broad absorption band centered at approximately 3393 cm^−1^ increased in intensity, which may be attributed to the overlap between the N-H stretching vibrations of the newly introduced amine groups and the intrinsic O-H stretching vibrations of the lignocellulosic matrix [20]. ARS exhibited enhanced absorption bands at 1644 and 1552 cm^−1^. The former is consistent with the N-H bending vibration of the newly introduced -NH_2_ groups, whereas the latter is primarily assigned to N-H deformation vibrations, potentially accompanied by C-N stretching vibrations [21]. Similar N-H-related absorption bands at approximately 1655 and 1565 cm^−1^ have also been reported in studies on aminated lignin [22]. The spectral variation observed at 1074 cm^−1^ may also include a contribution from C–N stretching vibrations. Collectively, the changes at 3393, 1644, and 1552 cm^−1^ provide evidence for the successful incorporation of amine-containing functional groups into the rice straw matrix. In the FT-IR spectrum of OSA, the absorption band at 1740 cm^−1^ was assigned to the C=O stretching vibration of aldehyde groups generated during the oxidation process, whereas the band at 800 cm^−1^ was associated with hemiacetal structures formed between the aldehyde groups and adjacent hydroxyl groups [23]. Compared with OSA, the intensities of both bands were markedly reduced in the ARS/OSA spectrum, indicating that the free aldehyde groups and the associated hemiacetal structures in OSA were consumed during its reaction with ARS. Meanwhile, the absorption intensity of ARS/OSA increased considerably at approximately 1614 cm^−1^, a spectral region characteristic of C=N stretching vibrations. This observation suggested that the amino groups in ARS may have undergone a condensation reaction with the aldehyde groups in OSA, resulting in the formation of Schiff-base linkages. Similarly, Genç et al. [24] reported that, in an oxidized alginate–gelatin system, the principal absorption band of oxidized alginate at 1597 cm^−1^ shifted to 1614 cm^−1^ and became broader after cross-linking with an amino-containing component. This spectral change was attributed to the overlap of the Schiff-base C=N stretching vibration with the absorption bands of the pre-existing functional groups. Compared with the spectra of OSA, there was a notable increase in peak intensity observed in the ARS/OSA spectra. Considering the potential replacement of C=O, it is possible that the emergence of C=N in the ARS/OSA sample could be responsible for this phenomenon. The spectra of ARS/OSA and iron-containing microspheres showed no significant difference, indicating that iron loading had no obvious impact on the main structure of ARS/OSA.

Figure 2b shows the X-ray diffraction (XRD) patterns of iron-containing microspheres and other samples. In the pattern of ARS, the peaks at 21.2° and 26.6° respectively correspond to the (002) and (040) crystalline planes of the cellulose 1β structure [25]. In the pattern of OSA, a characteristic peak at 13° corresponds to the amorphous structure [26]. Compared with the aforesaid two samples, the diffraction intensity of the peaks in the iron-containing microspheres pattern was notably weakened, which indicated that the relative crystallinity of cellulose in the iron-containing microspheres was lower [27]. This phenomenon could be attributed to the reduction in intermolecular and intramolecular hydrogen bonds due to the cross-linking reaction either between iron ions and polymer chains or between ARS and OSA [28]. XRD pattern of iron-containing microspheres exhibited several new diffraction peaks at approximately 30.0°, 35.4°, 37.6°, 42.8°, 54.0°, 57.8°, and 62.4°, which were indexed to the (220), (311), (222), (400), (422), (511), and (440) crystal planes of cubic γ-Fe_2_O_3_, respectively [29]. These diffraction peaks were in good agreement with the standard PDF/JCPDS card for γ-Fe_2_O_3_ (PDF#39-1346). These results indicate the formation of an Fe(III)-containing crystalline phase in the sample, thereby confirming the successful incorporation of iron into the ARS/OSA matrix.

The XPS survey of iron-containing microspheres and other samples is presented in Figure 2c. The peak corresponding to Na 1s emerged in the spectra of OSA [30]. In contrast to the spectra of OSA, the Na 1s peak disappeared in the spectra of iron-containing microspheres, which indicated that the sodium element in the sodium carboxylate groups might have been substituted by other components. The binding energy within the range of 700–740 eV was attributed to Fe 2p, indicating that the iron ions were probably bound to carboxylate groups through interionic interactions. The fitted plot of the iron peak in iron-containing microspheres is shown in Figure 2d. The curve-fitting results showed that the Fe 2p spectrum could be deconvoluted into six components centered at 709.5, 710.8, 714.2, 722.7, 724.1, and 727.6 eV. The peaks at 710.8 and 724.1 eV were assigned to the characteristic Fe 2p_3/2_ and Fe 2p_1/2_ signals of Fe(III), respectively [31,32], whereas those at 714.2 and 727.6 eV were attributed to Fe(III)-related high-binding-energy shoulders or multiplet-splitting contributions [33,34]. The weak peaks at 709.5 and 722.7 eV were ascribed to a minor fraction of Fe(II) or other low-binding-energy iron species. Semi-quantitative analysis based on the fitted peak areas revealed that Fe(III)-related signals accounted for approximately 92.0% of the total fitted area. These results suggested that iron in the iron-containing microspheres may exist predominantly in the Fe(III) oxidation state. This finding is consistent with the characteristic γ-Fe_2_O_3_ diffraction peaks observed in the XRD pattern. The O 1s scan of OSA is shown in Figure 2e. The peaks at 531.6 eV, 532.9 eV, and 535.6 eV corresponded to C−O, C=O, and sodium salt, respectively [35]. The results indicated that sodium alginate was successfully oxidized to sodium alginate oxide, which is consistent with the FT-IR analysis. The N 1s scan of ARS and iron-containing microspheres is presented in Figure 2f. The peaks at 401.9 eV and 399.8 eV were detected in the scan of ARS, which respectively pertain to the characteristic peak of the amino (R−NH_2_) groups and substituted amine (R−NH−R) [36]. These signals indicated the introduction of amino-containing groups into the rice straw matrix after amination. In addition, the relative intensity of the amine-related N 1s component decreased after reaction with OSA, suggesting that amino-containing groups participated in the formation of the ARS/OSA network. This result is consistent with the FT-IR spectral changes discussed above.

**Figure 3:**
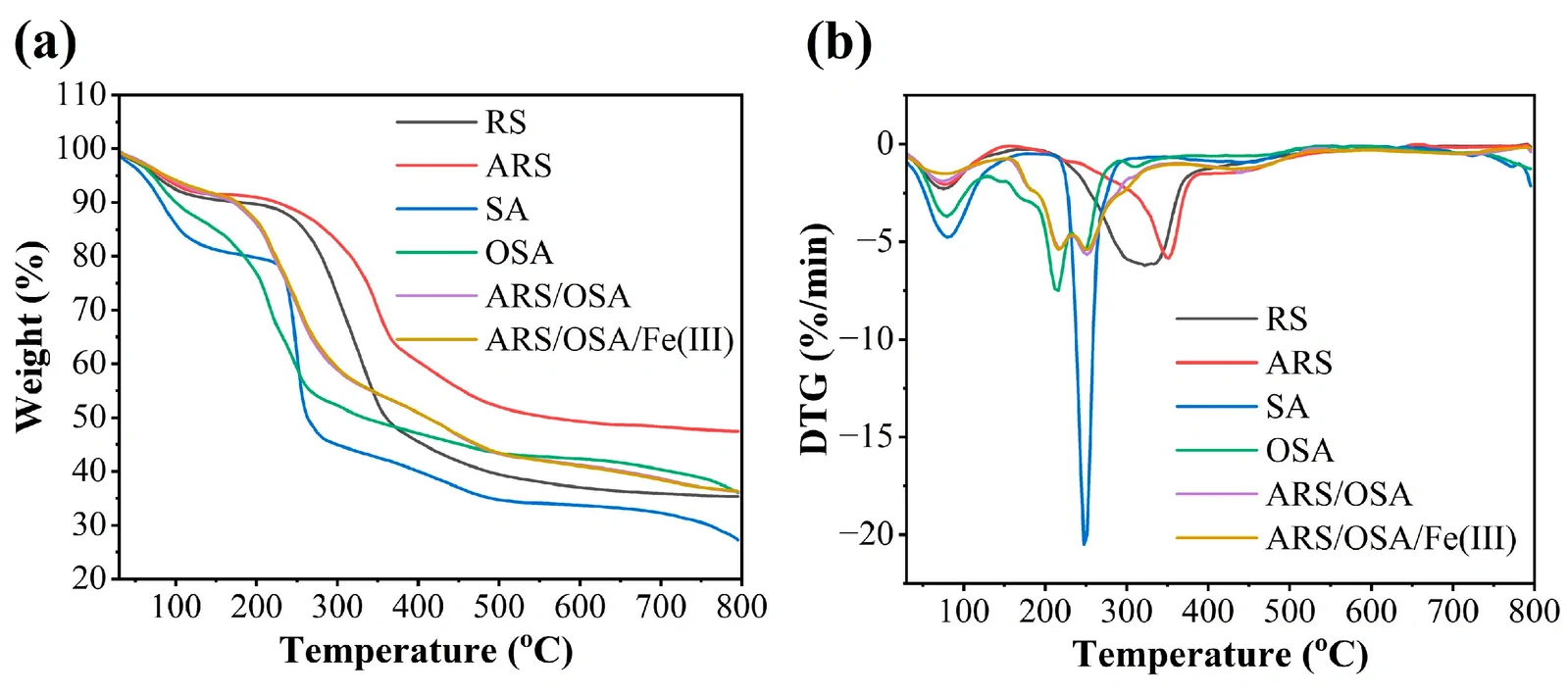
Thermogravimetric analysis (TGA) (**a**) and differential thermogravimetric (DTG) (**b**) curves of iron-containing microspheres and other samples.

Figure 3a,b show the TGA and DTG curves of iron-containing microspheres and other samples. In the temperature range of 50 °C to 150 °C (corresponding to the water evaporation stage) [37], both the RS and ARS samples showed slight mass loss, but no substantial difference was evident in the alteration of mass loss. From 250 °C to 350 °C, the mass loss rate of ARS was significantly lower compared to that of the ball-milled RS fine powder, and the maximum value for the mass loss rate of ARS emerged later, indicating that the thermal stability of ARS surpassed that of ball-milled RS fine powder. Pan et al. [38] found that grafting primary and secondary amines into the molecular structure of lignin could enhance the thermal stability of lignin macromolecules. Chen et al. [39] proposed that the pyrolysis of bamboo lignocellulose initiated from the branching functional groups within the temperature range of 250 °C to 350 °C. Ge et al. [40] demonstrated that the hydroxyl group with an enol-like structure in lignin can offer hydrogen atoms under an alkaline reaction condition and thereby be utilized to fix the grafted amine functional groups. The above-mentioned findings indicated that the improved thermal stability of ARS might be related to the structural changes in rice straw after amination. In the range of 300 °C to 350 °C, lignin in biomass can be pyrolyzed to form phenols and other substances by breaking ether bonds, while the temperature range of 450 °C to 650 °C corresponded to the biomass carbonization temperature [41]. Once the heating temperature reached 350 °C, no remarkable disparity in the change in mass loss rate was observed between the two samples with the increase in temperature, suggesting that the main thermal degradation behavior of the rice straw matrix was largely retained after amination.

In the temperature range of 150 °C to 230 °C, the mass loss rate of OSA was higher than that of sodium alginate (SA), and in the temperature range of 190 °C to 240 °C, the mass loss of OSA was greater than that of SA. The results indicated that the thermal stability of OSA was lower than that of SA. Ku et al. [42] discovered that after the SA underwent ring-opening, oxidation, and other reactions, its pyrolysis temperature would decrease. Wang et al. [43] also found that the thermal stability of sodium alginate with open-loop structural units decreased. Therefore, the thermal analysis results could verify that the oxidation reaction might have occurred in SA to form OSA. When the temperature was lower than 240 °C, the mass loss and mass loss rate of ARS/OSA were lower than those of OSA, suggesting that the combination of ARS and OSA enhanced the thermal stability of the new material. Li et al. [44] discovered that the fertilizer produced through a cross-linking reaction exhibited a denser structure compared to blended fertilizers, resulting in the polymer chain segment movement requiring more external energy. Therefore, it is possible that a chemical connection exists between OSA and ARS. Additionally, the TG and DTG curves of ARS/OSA and iron-containing microspheres are essentially coincident, indicating that the cross-linking of iron did not alter the basic structure of ARS/OSA.

**Figure 4:**
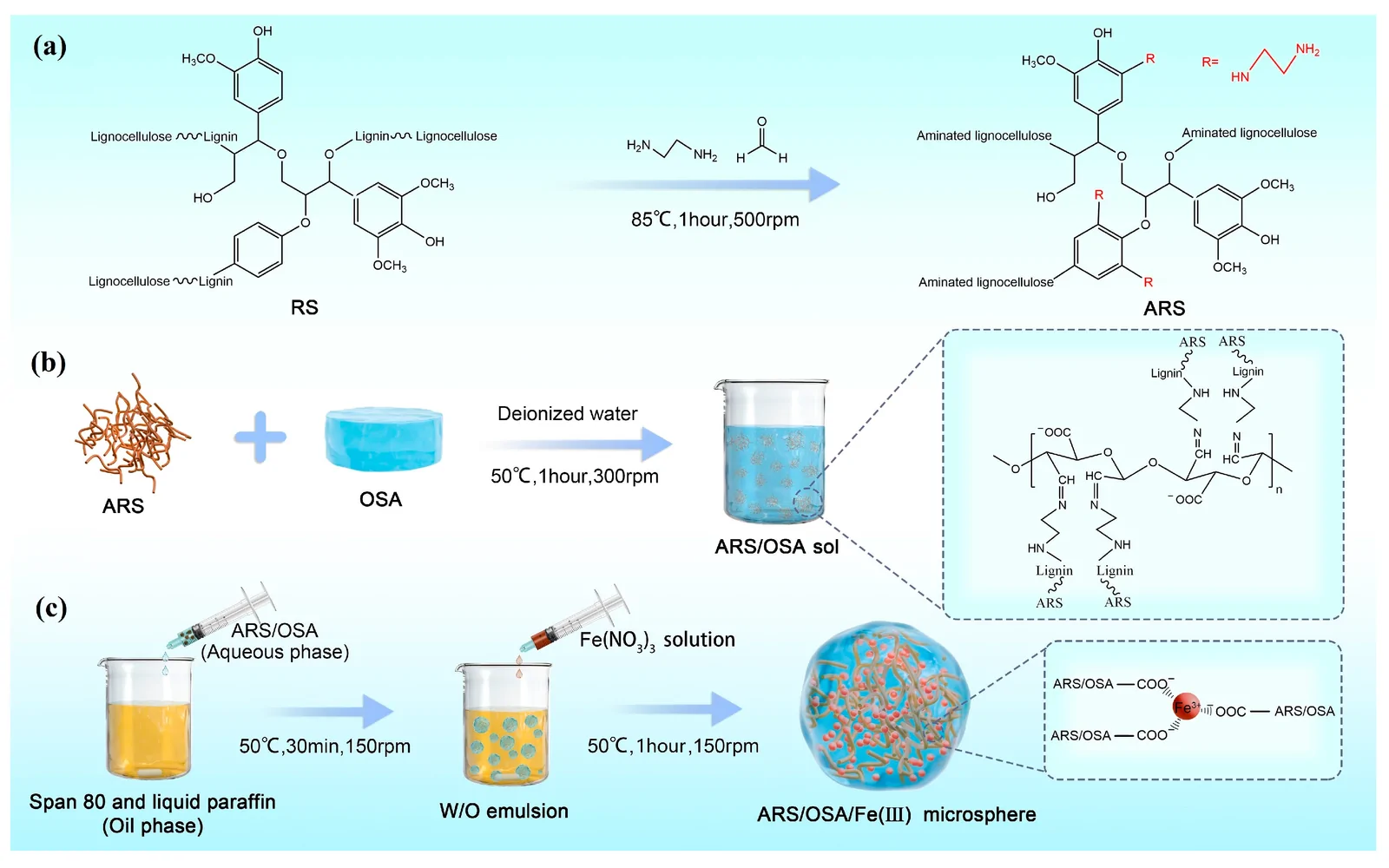
Proposed preparation pathway and possible interactions involved in the formation of iron-containing microspheres: synthesis of ARS (**a**); synthesis of ARS/OSA sol (**b**); synthesis of iron-containing microspheres (**c**).

To summarize, Figure 4 illustrates a proposed preparation pathway of the iron-containing microspheres. Ethylenediamine was used to introduce amino-containing groups into the rice straw matrix, and ARS was formed after amination treatment (Figure 4a). The subsequent reaction between ARS and OSA was proposed to involve interactions between amino-containing groups in ARS and aldehyde-related groups in OSA, leading to the formation of an ARS/OSA network (Figure 4b). After emulsification, Fe(III) was introduced into the microspheres and associated with carboxyl groups mainly through ionic interactions (Figure 4c).

### 2.3. Slow-Release Performance of the Iron-Containing Microspheres

**Figure 5:**
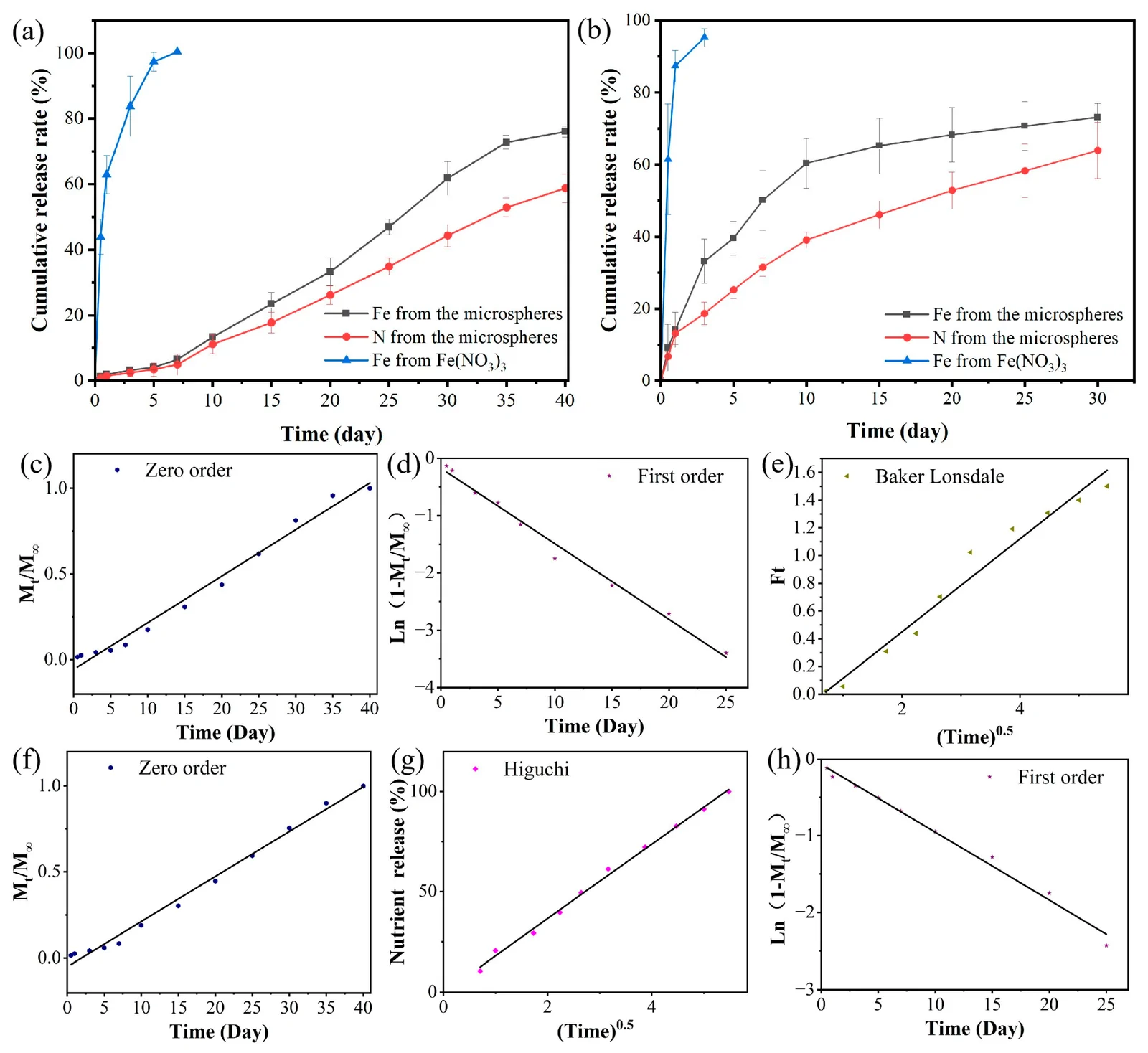
The nutrient release rate of iron-containing microspheres in soil column leaching test (**a**) and static water release test (**b**); the model with a high fitting degree for the release rule of iron element in the soil (**c**) and water (**d**,**e**); the model with a high fitting degree for the release rule of nitrogen element in the soil (**f**) and water (**g**,**h**).

The release rates and kinetic fitting curves of nutrients from iron-containing microspheres are shown in Figure 5 (data in Table S3). In the soil test, the release rates of the iron element at 1 day, 30 days, and 40 days were 1.94%, 61.8%, and 76.03%, respectively. In contrast, the 1-day iron release rate of Fe(NO_3_)_3_ as a control reached 62.91%. Therefore, the iron-containing microspheres hold potential for further development as an iron slow-release fertilizer. The release rate of nitrogen in the iron-containing microspheres was slower than that of the iron element, with the release rates reaching 1.47% and 44.32% on the first day and the thirtieth day, respectively. This phenomenon is most likely attributed to the fact that the iron element was fixed on OSA, whereas the nitrogen element was fixed on the lignocellulosic skeleton of RS during the amination process. Although covalent bonding may exist between OSA and the lignocellulosic framework, OSA exhibits hydrophilic properties. Iron was released concomitantly with the dissolution of OSA, while nitrogen release was contingent upon the degradation of the lignocellulosic framework. The degradation rate of lignocellulose was significantly slower than that of OSA. Kinetic analysis showed that the release profiles of both iron and nitrogen elements were well described by the zero-order model, with R^2^ values of 0.9852 and 0.9912, respectively. This indicates that the cumulative release of both nutrients increased approximately linearly with time, exhibiting relatively stable and sustained release characteristics [45]. Such release behavior may result from the combined effects of relatively slow water transport in soil, progressive hydration of the microspheres, structural changes in the matrix, nutrient desorption and diffusion, and interactions between the released nutrients and soil particles. For the early release stage, fitting with the Korsmeyer–Peppas model yielded release exponent (n) values of 0.884 and 0.940 for iron and nitrogen elements in soil, respectively, with corresponding R^2^ values of 0.9239 and 0.9383. Considering the approximately spherical morphology of the microspheres, these n values were preliminarily interpreted using the reference thresholds established for spherical systems. Because both n values exceeded 0.85, the early release behavior was consistent with Super Case II transport [46]. This result suggests that, in addition to nutrient diffusion, polymer-network relaxation, swelling, and structural rearrangement of the matrix may also contribute to the early-stage release of iron and nitrogen elements in soil.

In the static water releasing test, the iron element exhibited release rates of 14.13% at 1 day and 73.1% at 30 days, while the nitrogen element showed release rates of 13.14% at 1 day and 63.88% at 30 days. The outcomes demonstrated that the iron-containing microspheres exhibited a slow-release effect in water, but their release behavior was dissimilar to that in soil. In the first 10 days, the rates of iron and nitrogen element release were relatively swift. After 10 days and 25 days, the release rates of both declined. Kinetic analysis showed that the iron release profile was better described by the first-order and Baker–Lonsdale models. The first-order kinetic behavior indicates that the apparent iron release rate was related to the amount of releasable iron remaining within the microspheres and may reflect the gradual decrease in the concentration gradient during the release process [47]. The Baker–Lonsdale model provides a semi-empirical description of solute transport from the interior of a spherical matrix to the surrounding medium. Taken together, the initial hydration, dissolution, or desorption of the more readily releasable iron fraction, followed by the outward migration of iron through the hydrated porous network, may jointly contribute to iron release in water. In comparison, the nitrogen release profile in water was well described by the Higuchi and first-order models. The agreement with the Higuchi model suggests that diffusion of nitrogen through the hydrated porous matrix may represent an important contribution to the release process, whereas the first-order behavior indicates that the release rate gradually decreased as the remaining releasable nitrogen fraction and the corresponding driving force declined. Fitting the early-release data (F = M_t_/M_∞_ ≤ 0.60) to the Korsmeyer–Peppas model yielded n values of 0.664 and 0.538 for iron and nitrogen elements, respectively, with corresponding R^2^ values of 0.9900 and 0.9653. Under the assumption of an approximately spherical geometry, both n values fall within the anomalous transport, or non-Fickian diffusion, range [48,49]. These results suggest that nutrient diffusion, polymer-network relaxation, and structural changes in the matrix may jointly contribute to the early-stage release process in water. The column and static water experiments have provided preliminary insights into the release patterns of iron-containing microspheres, while comparative analysis of blank microspheres, non-ammonified materials, and sheet-shaped materials requires further in-depth study.

### 2.4. Water Absorption Performance of the Iron-Containing Microspheres

**Figure 6:**
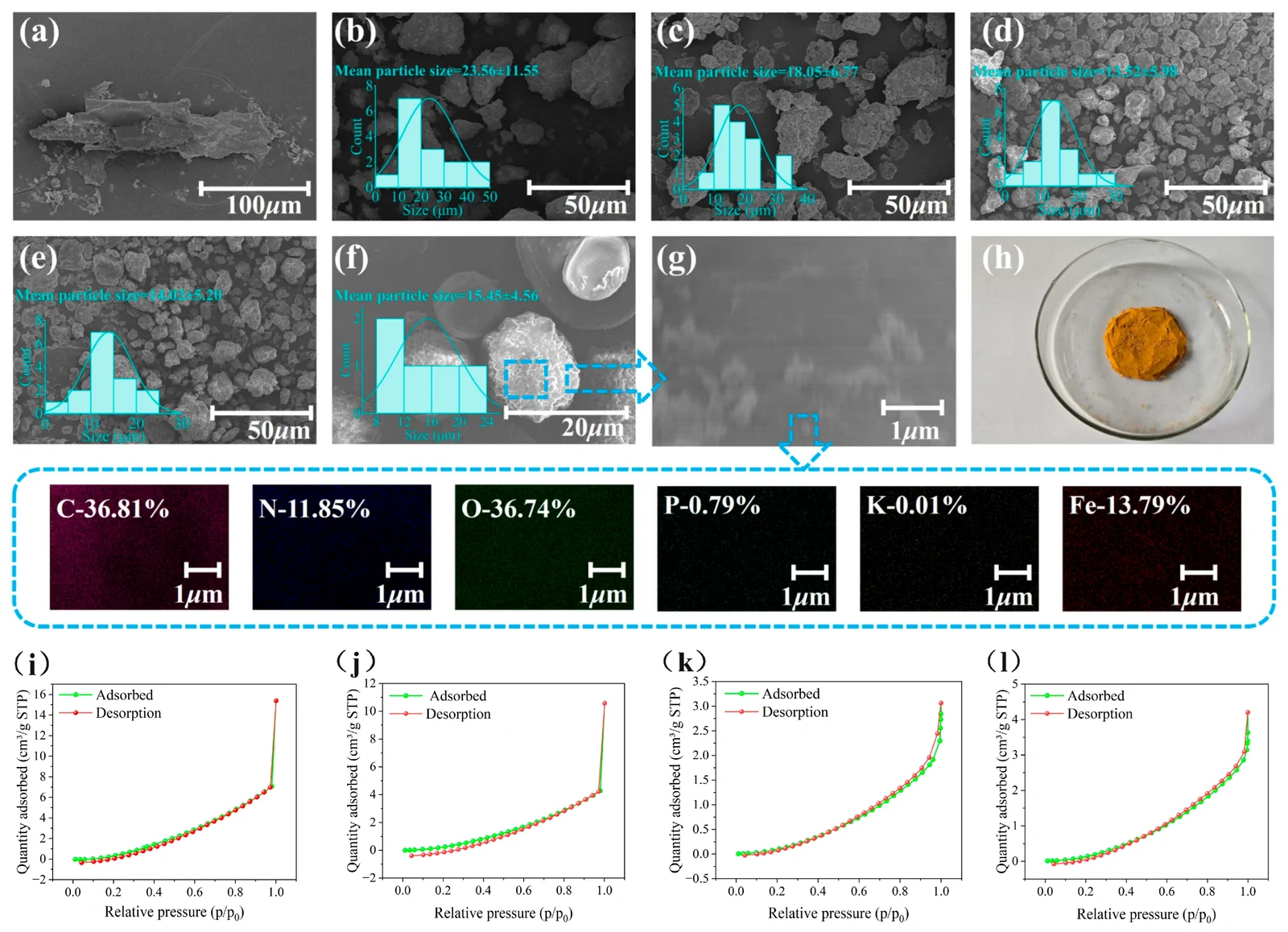
SEM of the rice straw powder (**a**); SEM of the ball-milled rice straw fine powder obtained by grinding for 4 h (**b**), 8 h (**c**), 12 h (**d**), and 16 h (**e**); EDS analysis of the iron-containing microspheres prepared from the ball-milled rice straw fine powder for 12 h (**f**,**g**); the photo of iron-containing microspheres (**h**); BET analysis of various iron-containing microspheres prepared from rice straw after grinding for 4 h (**i**), 8 h (**j**), 12 h (**k**), and 16 h (**l**).

The scanning electron microscopy (SEM) images of ball-milled rice straw fine powder with different grinding times and the energy-dispersive X-ray spectroscopy (EDS) analysis of iron-containing microspheres are presented in Figure 6. The water absorption rate and iron content of iron-containing microspheres prepared from ball-milled rice straw fine powder with different particle sizes are shown in Table 3. As indicated in Figure 6, when the grinding time reached 12 h, the particle size of the fine powder of rice straw approximated 13.5 μm. With the prolongation of the grinding time, the average particle size of the ball-milled rice straw sample ceased to decrease. As shown in Table 3, the disparity in iron content of the iron-containing microspheres prepared from ball-milled rice straw fine powder with different particle sizes was not significant, but the difference in water absorption rate was extremely significant. The water absorption capacity increased with the decrease in particle size and the increase in Brunauer–Emmett–Teller (BET) specific surface area and pore parameters. The Pearson correlation coefficients between water absorption and particle size, BET specific surface area, Barrett–Joyner–Halenda (BJH) pore volume, and BJH average pore size were −0.9999, 0.9755, 0.7689, and 0.9986, respectively. A descriptive Pearson correlation analysis showed that water absorption was negatively correlated with particle size and positively correlated with BET specific surface area, BJH pore volume, and BJH average pore size. Therefore, sample 3 and 4 achieved a water absorption rate of approximately 500%, which was significantly higher than that of the other samples. Compared with our previously reported sheet-shaped material, the present work converts the sheet-shaped system into microspheres through ball milling-assisted particle-size regulation and emulsion separation. This structural change improved the water-accessible structure, increasing the water absorption capacity from 97.83% to 501.76% and reducing the first-day iron release in water from 48.37% to 14.13% [11]. These results highlight the advancement of the present microsphere system in water absorption and delayed iron release.

In Figure 6f, the SEM image demonstrated that the iron-containing microspheres exist in the form of microspheres, featuring a rough surface and numerous small pores. This structural trait enables water molecules to permeate the microbeads, thereby facilitating rapid water absorption. In Figure 6g, the results of the EDS analysis indicated that oxygen, nitrogen, carbon, phosphorus, potassium, and iron were homogeneously distributed on the surface of the iron-containing microspheres (data in Figure S1 and Table S5). The EDS results indicated that the microspheres contained 13.79% iron, suggesting that the iron was loaded on the surface of the microspheres. The photo of iron-containing microspheres is presented in Figure 6h, where the Petri dish has a diameter of 9 cm. The nitrogen adsorption–desorption isotherms are shown in Figure 6i–l. The nitrogen adsorption–desorption isotherms of the microspheres prepared from straw samples with different particle sizes exhibited adsorption–desorption hysteresis in the medium-to-high relative-pressure region. In addition, the BJH average pore diameters of the representative samples ranged from 5.42 to 5.80 nm, which falls within the conventional mesoporous range of 2–50 nm. These results suggest the presence of mesoporous domains in the microsphere structures. Microspheres prepared from straw samples of different particle sizes all exhibit typical mesoporous structural characteristics [50]. The ball milling process can accelerate the breakdown of cell wall structures, improve the pore structure, and promote the formation of new mesoporous structures in the wood fibers [51]. As the particle size of rice straw decreased, the representative BET results showed general increases in the specific surface area and BJH adsorption pore volume, while the BJH average pore diameter remained within a relatively narrow range, suggesting possible development of the internal pore structure. The larger specific surface area and pore volume provide more space for water adsorption and storage, while the well-developed mesoporous structure facilitates the diffusion and transport of water within the material, thereby enhancing the microspheres’ water absorption performance [52]. The structures and properties of some representative bio-based, alginate, and hydrogel-based sustained-release fertilizers are shown in Table 4. Compared with these studies, the iron-containing microspheres integrate the water absorption property and the slow-release properties of iron and N in the same system. Particularly, compared with the previously reported thin sheet-shaped materials, the microsphere structure enhances the water absorption capacity of the material.

### 2.5. Pot Culture Experiments

**Figure 7:**
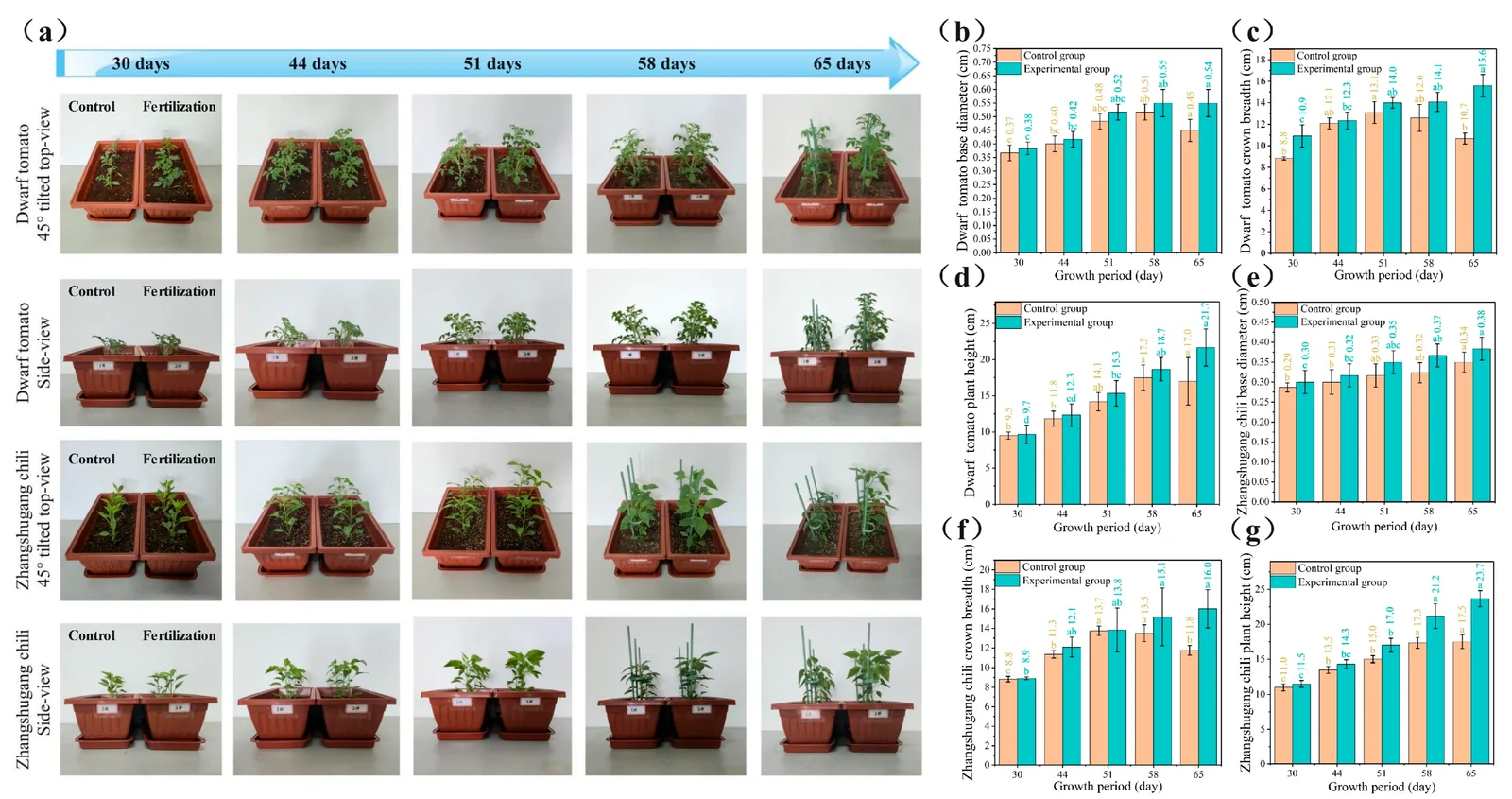
(**a**) Photos of dwarf tomatoes and Zhangshugang chili seedlings from days 30–65; the basal diameter (**b**), canopy diameter (**c**), and plant height (**d**) of dwarf tomato; the basal diameter (**e**), canopy diameter (**f**), and plant height (**g**) of Zhangshugang chili.

Photographs of the potted trials of dwarf tomato and Zhangshugang chili seedlings are shown in Figure 7a. The base diameter, canopy diameter, and plant height are shown in Figure 7b–g. Compared with the control group, plants treated with iron-containing microspheres exhibited better growth characteristics between 30 and 65 days. By day 65, the seedlings in both control groups had shown significant wilting. After irrigation was stopped, the canopy diameter of the dwarf tomato plants in the control groups decreased by day 51, while the stem diameter and plant height decreased by day 65. In the control group, there was no significant decrease in the base diameter or plant height of the Zhangshugang chili seedlings, but the canopy diameter decreased significantly. Compared with the control group, the aforementioned indicators for dwarf tomato seedlings and Zhangshugang chili seedlings treated with microsphere-based controlled-release fertilizer all showed an increasing trend. The results showed that seedlings treated with iron-containing microspheres exhibited better growth compared to the control group, and no significant wilting was observed even by day 65. However, the underlying mechanisms by which the iron-containing microspheres affect plant growth require further in-depth investigation.

## 3. Conclusions

In this study, iron-containing microspheres possessing water absorption and slow-release functions were prepared. The amination reaction of lignin was utilized to enhance the reactivity of rice straw lignocellulose. The comprehensive analysis results indirectly suggested that a Schiff’s base cross-linking reaction likely occurred between aminated rice straw and oxidized sodium alginate. This approach effectively retards the dissolution rate of oxidized alginate in water, thereby achieving a slow release of iron and nitrogen elements. The ionic interaction between oxidized alginate and iron ions has enhanced the iron loading capacity, resulting in iron-containing microspheres with an iron content of up to 10.44%. Meanwhile, the ball milling and emulsion separation processes have contributed to the formation of a distinctive microsphere structure for iron-containing microspheres, leading to an extremely high water absorption capacity of up to 501.76%. The iron-containing microspheres have the potential to be further developed into slow-release fertilizers. Future research should prioritize green production and cost reduction, particularly addressing issues related to solvent residue, solvent recovery, and energy consumption, to transition the preparation of iron-containing microspheres from laboratory scale to large-scale production.

## 4. Materials and Methods

### 4.1. Material

The air-dried rice straw was harvested from Lianyungang City, Jiangsu Province, China. It was pulverized using a plant cell crusher (FW-100, Tianjin Taisite Instrument Co., Tianjin, China) and subsequently sieved to collect the powder in the 60–100 mesh size range. Following this, the powder underwent Soxhlet extraction with absolute ethanol for 24 h. The extracted rice straw was dried at 105 °C for 6 h. The contents of cellulose, hemicellulose, lignin, and ash in dried rice straw are 48.4%, 18.6%, 17.8%, and 15.2%, respectively. Sodium hydroxide (96%), ethylenediamine (EDA, 99%), ferric nitrate (Fe(NO_3_)_3_·9H_2_O, 98%), sorbitan oleate (Span 80, 98%), and liquid paraffin (97%) were purchased from Sinopharm Chemical Reagent Co., Ltd. (Shanghai, China). The purity of formaldehyde solution (37 wt% in H_2_O), sodium periodate, and ethylene glycol met ACS reagent standards (Aladdin Biochemical Technology Co., Ltd., Shanghai, China).

### 4.2. Preparation Method of Iron-Containing Microspheres

Each 500 mL nylon grinding jar was filled with 15 g of rice straw powder and approximately 200 g of zirconium dioxide grinding balls (3 mm in diameter). Four such jars were secured in a DECO-PBM-V-2L planetary ball mill (Changsha DECO Instrument Equipment Co., Ltd., Changsha, China) for the operation. The speed ratio between the large plate and the milling vessel was set at 1:2, with the revolution speed of the large plate fixed at 350 rpm. The ball mill underwent a 5 min stoppage every 10 min during operation. The total grinding time, including operating and pause periods, was set at intervals of 4, 8, 12, and 16 h.

After completion of the ball milling procedure, 8 g of ball-milled rice straw fine powder, a mixture containing 200 mL of 0.01 mol·L^−1^ NaOH solution and 10 mL of EDA was prepared. The mixture was heated at 85 °C for 30 min using a magnetic stirring heater (MS7-H550-Pro, DLAB Scientific Inc., Beijing, China). A formaldehyde solution (10 mL) was dropped into the aforesaid mixture over a period of 10 min. The reaction was allowed to continue under continuous heating and agitation for another 1 h. Subsequently, an HCl solution (0.1 mol·L^−1^) was employed to adjust the pH of the mixture to 4. Following this, the reaction mixture underwent vacuum filtration. After approximately 30 mL of distilled water was employed to wash the solid residue, it was dried at 105 °C until reaching constant weight to obtain the aminated rice straw (ARS). The amino group content of ARS measured by chemical titration was 1.81 mmol·g^−1^, corresponding to a calculated amination degree of 2.53%. The oxidized sodium alginate (OSA) was synthesized by the oxidation reaction of sodium alginate with sodium periodate as detailed in our previous study [11]. Briefly, sodium alginate (20 g), sodium periodate (6.5 g), and 50% (*v*/*v*) aqueous ethanol solution (200 mL) were mixed and stirred in the dark at room temperature for 6 h. Ethylene glycol (6.5 g) was added to the mixture to terminate the reaction. After centrifugation at 3000 rpm, the precipitate was washed with a 50% (*v*/*v*) ethanol solution. The washing and centrifugation process was repeated three times. The washed precipitate was dried under vacuum at 45 °C to obtain OSA. The theoretical oxidation degree of OSA was calculated to be approximately 30 mol% based on the molar ratio of sodium periodate to alginate sodium repeating units.

The OSA and ARS were mixed at a designated OSA-to-ARS mass ratio, which was recorded as X_1_, and then added to 100 mL of deionized water to obtain the ARS/OSA sol as the aqueous phase. The mixture was heated at 50 °C for 1 h to obtain the ARS/OSA sol as an aqueous phase. The mass ratio of the combined ARS and OSA to the deionized water was recorded as X_2_. Span 80 and liquid paraffin were mixed at a volume ratio of 1:20 and heated at 50 °C for 30 min to obtain the oil phase. While maintaining the oil phase at 50 °C and stirring at 150 rpm, a designated volume of the previously prepared aqueous-phase sol was subsequently added. The total volume of aqueous phase sol and oil phase combined equaled 200 mL, and their volume ratio was recorded as X_3_. The system continued to be heated and stirred for another 30 min to form the W/O emulsion. Subsequently, a Fe(NO_3_)_3_ aqueous solution (50 mL) was added to the W/O emulsion, with its weight concentration recorded as X_4_. After being further heated and agitated for 1 h, a total volume of 300 mL anhydrous ethanol and n-hexane (in a volumetric ratio of 1:1) was introduced into the system, after which a further 15 min of agitation prior to vacuum filtration took place. The solid residue obtained from this process underwent lyophilization to yield the iron-containing microspheres. A central composite experiment with four factors and five levels was conducted to determine the optimal preparation conditions for iron-containing microspheres, as detailed in Table 1. The data were analyzed using the Design Expert 13 software (Stat-Ease, Inc., Minneapolis, MN, USA).

### 4.3. Characterization Method of Iron-Containing Microspheres

The iron content in iron-containing microspheres was determined using an iCE3500 atomic absorption spectrophotometer (Thermo Fisher Scientific Inc., Waltham, MA, USA). The nitrogen content was analyzed with a KN680 Kjeldahl nitrogen analyzer (Jinan Alva Instrument Co., Jinan, China). FT-IR spectra of different samples were recorded on a Nicolet iS 20 spectrometer (Thermo Fisher Scientific Inc., Waltham, MA, USA). The scanning range was from 4000 cm^−1^ to 500 cm^−1^. The resolution and scan number were 2 cm^−1^ and 16 times, respectively. Thermogravimetric (TG) and Derivative Thermogravimetry (DTG) analysis of iron-containing microspheres and other samples were conducted using a NETZSCH STA 449 F5 thermal analyzer (NETZSCH Scientific Instruments Co., Ltd., Selb, Germany). The temperature rise rate was set at 10 °C·min^−1^. XPS measurements were performed using a Thermo Scientific Nexsa X-ray photoelectron spectrometer (Thermo Fisher Scientific, East Grinstead, UK) equipped with a monochromatic Al Kα source operated at 150 W, with a photon energy of 1486.6 eV. The XPS data were processed and fitted using Avantage software (version 5.9921). All binding energies were charge-corrected by setting the C–C/C–H component of the C 1s spectrum to 284.8 eV. Before peak fitting, the background of each high-resolution spectrum was subtracted using the Smart background function, and peak deconvolution was performed using Gaussian–Lorentzian mixed line shapes. The Fe 2p spectrum was fitted using a constrained fitting procedure, in which the peak positions, full widths at half maximum, and area relationships of the individual components were optimized under predefined constraints. The fitting quality was evaluated using R^2^, and an R^2^ value of 0.883 was obtained. XRD patterns from various samples were obtained using an X-ray diffractometer (Rigaku SmartLab SE, Rigaku Corporation, Osaka, Japan) with Cu-K*α* radiation. The scanning speed was set at 2°·min^−1^ with a 2θ range of 5–90°. The N_2_ adsorption–desorption isotherms of the samples were measured using a specific surface area and pore size analyzer (BELSORP-mini II, BEL Japan Inc., Osaka, Japan). The test was conducted at 77 K, and the samples were subjected to a 6 h vacuum degassing process. The microstructure and surface composition of the samples were analyzed using a GeminiSEM 300 scanning electron microscope (SEM, Carl Zeiss AG, Oberkochen, Germany). All the samples were subjected to gold plating treatment.

### 4.4. Performance Evaluation of Iron-Containing Microspheres

#### 4.4.1. Release Performance Analysis

The release performance of the iron-containing microspheres in soil and water was assessed through the soil column leaching test and static water release test, respectively. The soil column leaching test was conducted using a polymethylmethacrylate tube with a diameter of 5 cm and a length of 25 cm. The amount of the added sample was set at 1 g. The detailed filling approach and test method have been elaborately presented in our previous study [11]. Briefly, two layers of sterile gauze were placed at the bottom of the tube, followed by sequential addition of quartz sand (25 g), soil (50 g), a mixture (50 g consisting of 49 g soil and 1 g iron-containing microspheres), and another layer of quartz sand (25 g). The packed column was compressed to approximately 12 cm in height, and 50 mL of distilled water was added dropwise to achieve slightly saturated moisture content. The experiment was conducted at room temperature. For each sampling, 50 mL of distilled water was added dropwise into the tube, and 50 mL of eluate was collected for analysis. The leaching process was carried out 11 times on days 1, 3, 5, 7, 10, 15, 20, 25, 30, 35, and 40. An iron nitrate control group was added to compare the release behavior of iron microspheres with that of soluble iron salts. Since the objective of the experiment was to preliminarily evaluate the release performance of iron-containing microspheres, soil nutrient content was not analyzed. The static water release test took place in a 250 mL screw-top bottle where 0.5 g of iron-containing microspheres and 100 mL of distilled water were combined at 25 °C. Following each extraction step involving the removal of every 10 mL of solution from the bottle, an equal volume (10 mL) of distilled water was added. The contents of iron and nitrogen in the eluent and solution were determined using an atomic absorption spectrometer (Thermo Fisher Scientific Inc., Waltham, MA, USA) and a Kjeldahl nitrogen analyzer (Jinan Alva Instrument Co., Jinan, Shandong, China), respectively. The particle size of ball-milled rice straw powder was determined by image-based analysis using ImageJ software (version 1.54g, National Institutes of Health, Bethesda, MD, USA). For each sample, at least 15 particles were measured from SEM images, and the results are presented as mean ± standard deviation. The release rate of nutrient elements was calculated based on the nutrient element contents as described in our previous study [11]. Specifically, the cumulative release rates in the water slow-release experiments were determined using Equation (3):

(3) $$E=\frac{V_{E}\sum_{1}^{n-1}C_{i}+V_{0}C_{n}}{m_{0}}\times 100$$

where *E* represents the cumulative release amount of iron (%) and N (%); *V_E_* and *V*_0_ are the sampling volume and initial volume (mL), respectively; *C_i_* and *C_n_* represent iron and N concentrations (mg/mL); *n* and *i* are the sampling time; and *m*_0_ is the iron and N content of the sample (mg). This was repeated three times for each sample and averaged.

Furthermore, the release rates in the soil slow-release experiments were calculated according to Equation (4):

(4) $$Release\;rate(\%)=\frac{CV}{m_{0}}\times 100$$

where *C* is the concentration of iron and N, mg/mL; *V* is the volume of solution (*V* = 50 mL); *m*_0_ is the content of iron and N in the initial fertilizer, mg. The tests were performed in triplicate. Data were presented as mean ± standard deviation.

#### 4.4.2. Water Absorption Determination

The water absorption percentage of the iron-containing microspheres was determined using a gravimetric method. Briefly, 2 g of dried iron-containing microspheres was accurately weighed. The sample was placed in a beaker containing 50 mL of deionized water and immersed at 25 °C for 1 h. After immersion, the hydrated microspheres were collected using 200-mesh gauze. Without applying external pressure, the sample was allowed to drain for 10 min to remove free water from the particle surfaces. The sample was then weighed immediately. The water absorption percentage was calculated using Equation (5):

(5) $$\mathrm{Water}\;\mathrm{absorption}\;\mathrm{rate}\;(\%)=\frac{m_{2}-m_{1}}{m_{1}}\times 100$$

where *m*_1_ represents the iron-containing microspheres mass; *m*_2_ represents the mass of filter residue.

#### 4.4.3. Pot Culture Experiment

Dwarf tomato and Zhangshugang chili seedlings that had been growing for 30 days were selected for a pot-grown experiment conducted in the laboratory. The seedlings were transplanted into pots measuring 35 cm in length × 19.0 cm in width × 14.5 cm in height, filled with equal amounts of dry soil. The soil was low-nutrient soil obtained from the Horticulture Station of Northeast Agricultural University. Each pot was planted with three seedlings. A total of 3 g of iron-containing microspheres was added to the experimental group. The control group received no fertilizer or external materials. A standardized watering schedule was used, watering once every 7 days with approximately 500 mL of water per irrigation. To preliminarily assess the impact of iron-containing microspheres on the seedlings, watering was stopped on the 21st day, and the growth conditions of different seedlings were observed. Measure the growth parameters of the seedlings every 7 days, including plant height, canopy diameter, and basal diameter, and take photographs.

#### 4.4.4. Statistical Analysis

All material characterization and release experiments were performed with three independent technical replicates unless otherwise stated. For the pot culture experiment, three biological replicates were used for each treatment, with each pot considered as one biological replicate. Data are presented as mean ± standard deviation. Statistical analysis was performed using SPSS 17.0 software (IBM Investment Co., Ltd., Shanghai, China). Differences among treatments were analyzed by one-way analysis of variance (ANOVA), followed by Duncan’s multiple range test at a significance level of *p* ≤ 0.05. The release kinetics of iron-containing microspheres were analyzed using mathematical models [53,54], and Origin 2023b software (OriginLab Co., Ltd., Northampton, MA, USA) was used to generate the fitting curves.

## Figures and Tables

**Table 1:** Experimental design and levels of the independent variables.

Variable	Coding	Coded Levels				
		−2	−1	0	1	2
Mass ratio of oxidized sodium alginate (OSA) to aminated rice straw (ARS) (g:g)	*X*_1_	1	2	3	4	5
Ratio of the total mass of ARS and OSA to the mass of deionized water (g:mL)	*X*_2_	2	3	4	5	6
Volume ratio of oil phase to aqueous phase (mL:mL)	*X*_3_	1	1.5	2	2.5	3
Weight concentration of Fe(NO_3_)_3_ solution (%)	*X*_4_	5	6	7	8	9

**Table 2:** Independent-batch validation of the optimized iron-containing microspheres formulation.

Response	Predicted Value	Experimental Value	95% CI	Relative Error
Iron content	10.75 wt%	10.44 ± 0.50 wt%	9.20–11.68 wt%	2.97%
Water absorption rate	511%	501.76 ± 17.94%	457.19–546.33%	1.84%

**Table 3:** The effect of the total grinding time on particle size of ball-milled rice straw fine powder, and the water absorption rate, iron content, and BET analysis results of iron-containing microspheres prepared from different ball-milled rice straw fine powder.

Sample	Grinding Time (Hour)	Particle Size (μm)	Water Absorption Rate (%)	Content of Iron (%)	BET Specific Surface Area (m^2^/g)	BJH Adsorption Pore Volume (cm^3^/g)	BJH Average Pore Size (nm)
1	4	23.56 ± 11.55 ^b^	308.87 ± 16.35 ^c^	9.69 ± 0.62 ^a^	0.6635	0.003466	5.4234
2	8	18.05 ± 6.77 ^b^	417.17 ± 24.33 ^b^	9.45 ± 0.67 ^a^	2.5621	0.006439	5.6199
3	12	13.52 ± 5.98 ^ab^	501.76 ± 17.94 ^a^	10.44 ± 0.50 ^a^	5.9103	0.010586	5.8046
4	16	14.02 ± 5.20 ^a^	493.12 ± 20.62 ^a^	10.17 ± 0.33 ^a^	5.9611	0.020790	5.7918

Note: Particle-size data are presented as the mean ± standard deviation based on measurements of 15 particles from the SEM images of each sample. All other values are expressed as the mean ± standard deviation (*n* = 3). Different lowercase letters within the same column indicate significant differences among samples at *p* < 0.05, whereas values sharing at least one letter are not significantly different.

**Table 4:** Comparison of the present iron-containing microsphere with representative bio-based, alginate, and hydrogel-based sustained-release fertilizers.

System	Matrix/Carrier	Structure	Reported Function	Difference or Advantage of the Present Work
Lignocellulose-based coating fertilizers [3,4]	Straw-derived or lignocellulosic materials	Coating layer	Delayed nutrient release through physical barrier or hydrophobic effect	The present system constructs an Fe-containing microsphere rather than only a coating barrier, and combines iron loading, water absorption, and Fe/N delayed release.
Hydrothermal carbonized straw materials [5]	Straw-derived carbon-rich materials	Porous carbonaceous structure	Soil improvement and environmental application	The present method retains the rice straw lignocellulosic framework and constructs microspheres without complete carbonization of straw.
Semi-interpenetrating hydrogel fertilizer CPAUH [10]	Carboxylated cellulose-based hydrogel	Semi-interpenetrating hydrogel network	Slow release of urea and humic acid over 15 days	The present material introduces iron loading into a rice straw/oxidized-alginate microsphere and shows coupled water absorption and Fe/N delayed release.
Previous ARS/OSA/Fe(III) fertilizer [11]	Aminated rice straw/oxidized sodium alginate	Sheet-shaped material	Controlled iron release, but limited water absorption	The present work converts the ARS/OSA/Fe(III) system into microspheres, increasing water absorption to 501.76 ± 17.94% and reducing the first-day iron release in water to 14.13%.
Present iron-containing microsphere (This study)	Aminated rice straw/oxidized sodium alginate	Microsphere with porous structure	Iron loading, water absorption, and Fe/N delayed release	Integrates rice straw-based microsphere construction, iron loading, high water absorption, and delayed Fe/N release in one material.

## Data Availability

The original contributions presented in this study are included in the article/Supplementary Materials. Further inquiries can be directed to the corresponding authors.

## Supplementary Materials

The following supporting information can be downloaded at https://www.mdpi.com/article/10.3390/gels12080700/s1, Figure S1: The distribution diagram total spectrum diagram for EDS analysis of the iron-containing microspheres; Table S1: Results of the central composite experiment for the preparation of iron-containing microspheres; Table S2: ANOVA analysis results of the quadratic response surface model of iron content and water absorption rate; Table S3: The analysis data of release kinetics of iron-containing microspheres in water and soil; Table S4: Korsmeyer–Peppas fitting of the early iron and nitrogen release from iron-containing microspheres; Table S5: EDS analysis data of the iron-containing microspheres.

## References

[1] Fan X.-R., Kong H.-Y., Zhao Y., Hu H.-Y., Jiang Z.-Q., Wang X.-L. (2026). Global synthesis of agricultural practices on crop yield, soil organic carbon and greenhouse gas emissions. J. Clean. Prod..

[2] Liu Q.-H., Bai C.-H., Zhang Z.-J., Yin X.-Y., Lin W.-T., Huang Y.-H., Yao L.-X. (2023). Straw incorporation induces rice straighthead disease in As-contaminated paddy soil. Sci. Total Environ..

[3] Thirumurugan N.K., Velu G., Murugaiyan S., Maduraimuthu D., Ponnuraj S., Sharmila D.J., Subramanian K.S. (2025). Nano-biofertilizers: Utilizing nanopolymers as coating matrix—A comprehensive review. Biofabrication.

[4] Boberski P., Główka M., Torchała K., Kulczycki G., Kuźnik N. (2025). Sustainable agriculture solutions: Biodegradable coatings for enhanced-efficiency fertilizers using cellulose and lignin. J. Agric. Food Chem..

[5] Ali T., Qiu P.-Y., Liu P., Muhammad N., Chen G.-Q., Fu T.-F., Peng C.-S. (2026). Transforming soil health with biochar-driven slow-release fertilizers: Emphasis on biochar dual-function for acidification and alkalinization reclamation. J. Environ. Chem. Eng..

[6] Ahmed M.J., Hameed B.H., Hummadi E.H. (2021). Insight into the chemically modified crop straw adsorbents for the enhanced removal of water contaminants: A review. J. Mol. Liq..

[7] Zhang Q., Yu G.-B., Zhou Q.-C., Li J.-C., Feng Y.-H., Wang L.-Z., Tang Y.-Y., Peng Y. (2020). Eco-friendly interpenetrating network hydrogels integrated with natural soil colloid as a green and sustainable modifier for slow release of agrochemicals. J. Clean. Prod..

[8] Bulut E. (2021). Development and optimization of Fe^3+^-crosslinked sodium alginate-methylcellulose semi-interpenetrating polymer network beads for controlled release of ibuprofen. Int. J. Biol. Macromol..

[9] Gao D.-P., Ran C., Zhang Y.-H., Wang X.-L., Lu S.-F., Geng Y.-Q., Guo L.-Y., Shao X.-W. (2022). Effect of different concentrations of foliar iron fertilizer on chlorophyll fluorescence characteristics of iron-deficient rice seedlings under saline sodic conditions. Plant Physiol. Biochem..

[10] Pan J.-X., Wang Y.-H., Wang J.-W., Qiu W.-W., Xiao Q., Dong S.-Q. (2026). CNF/p(AM-co-KAA) semi-interpenetrating network hydrogel fertilizer carriers for enhanced nutrient use efficiency, water retention, and salt–alkali resistance. Gels.

[11] Zhu C.-H., Zhang S.-M., Yi C., Heng Z.-Y., Wang Z.-J., Liu C.-H., Zheng X.-Z. (2023). Aminated rice straw/oxidized sodium alginate/iron(III): Synthesis and slow-release properties of a biomass-based material used as base fertilizer. Ind. Crops Prod..

[12] Wu X.-D., Sun L.-N., Qin B.-Y., Wang T., Wang Y., Zhao J.-R., Fu Y.-J. (2024). Novel antimicrobial polysaccharide hydrogel with fertilizer slow-release function for promoting *Sesamum indicum* L. seeds germination. Polymer.

[13] Li Y., Yuan W., Li L.-C., Miao R., Dai H., Zhang J.-H., Xu W.-F. (2020). Light-dark modulates root hydrotropism associated with gravitropism by involving amyloplast response in *Arabidopsis*. Cell Rep..

[14] Jeong J., Merkovich A., Clyne M., Connolly E.L. (2017). Directing iron transport in dicots: Regulation of iron acquisition and translocation. Curr. Opin. Plant Biol..

[15] Liang X.-X., Chen S.J., Liang Y.-X., Wang M.-M., Wang Q., Chen D.-X., Ma X., Ding H.-Y., Zhong H.-J. (2026). Alginate-based hydrogels: Recent progress in preparation, property tuning, and multifunctional applications. Gels.

[16] Zhang R.-Y., Yang L., Li L., Huang H.-Y., Song Y., Lei Y.-C., Hu Y., Chen S.-Y., Liu F. (2026). Advance in the characteristics, bioactivities, and drug delivery application of chitosan and its derivatives for osteoarthritis: A review. Carbohydr. Polym..

[17] Das S.K. (2025). Aminated biomass straw/oxidized sodium alginate/nutrients microsphere: Enhancing water absorption and controlled release fertilizer of bio-based substances. Waste Biomass Valor..

[18] Gao C., Wang X.-L., An Q.-D., Xiao Z.-Y., Zhai S.-R. (2021). Synergistic preparation of modified alginate aerogel with melamine/chitosan for efficiently selective adsorption of lead ions. Carbohydr. Polym..

[19] Yu C., Zhou W., Liu H., Liu Y., Dionysiou D.D. (2016). Design and fabrication of microsphere photocatalysts for environmental purification and energy conversion. Chem. Eng. J..

[20] Jiao G.-J., Peng P., Sun S.-L., Geng Z.-C., She D. (2019). Amination of biorefinery technical lignin by Mannich reaction for preparing highly efficient nitrogen fertilizer. Int. J. Biol. Macromol..

[21] Liu L.-Y., Wan X., Chen S.-W., Boonthamrongkit P., Sipponen M., Renneckar S. (2023). Solventless Amination of Lignin and Natural Phenolics using 2-Oxazolidinone. ChemSusChem.

[22] Gao S.-H., Zhu C.-Z., Ma L.-F., Liu C.-H., Zhang H.-Q., Zhang S.-M. (2023). Preparation of an Aminated Lignin/Fe(III)/Polyvinyl Alcohol Film: A Packaging Material with UV Resistance and Slow Release Function. Foods.

[23] Chi J.-H., Li A., Zou M.-Y., Wang S., Liu C.-Q., Hu R., Jiang Z.-W., Liu W.-S., Sun R.-J., Han B.-Q. (2022). Novel dopamine-modified oxidized sodium alginate hydrogels promote angiogenesis and accelerate healing of chronic diabetic wounds. Int. J. Biol. Macromol..

[24] Genç H., Hazur J., Karakaya E., Dietel B., Bider F., Groll J., Alexiou C., Boccaccini A.R., Detsch R., Cicha I. (2021). Differential Responses to Bioink-Induced Oxidative Stress in Endothelial Cells and Fibroblasts. Int. J. Mol. Sci..

[25] Thai Q.B., Nguyen S.T., Ho D.K., Tran T.D., Huynh D.M., Do N.H.N., Luu T.P., Le P.K., Le D.K., Phan-Thien N. (2020). Cellulose-based aerogels from sugarcane bagasse for oil spill-cleaning and heat insulation applications. Carbohydr. Polym..

[26] Cui S., Zhang S., Coseri S. (2022). An injectable and self-healing cellulose nanofiber-reinforced alginate hydrogel for bone repair. Carbohydr. Polym..

[27] Zhang A., Zou Y., Xi Y., Wang P., Zhang Y., Wu L., Zhang H. (2021). Fabrication and characterization of bamboo shoot cellulose/sodium alginate composite aerogels for sustained release of curcumin. Int. J. Biol. Macromol..

[28] Yan Y., Guan S., Wang S., Xu J., Sun C. (2022). Synthesis and characterization of protocatechuic acid grafted carboxymethyl chitosan with oxidized sodium alginate hydrogel through the Schiff’s base reaction. Int. J. Biol. Macromol..

[29] Li A., Liu Y.-X., Xu X.-J., Zhang Y.-Y., Si Z.-C., Wu X.-D., Ran R., Weng D. (2020). MOF-derived (MoS_2_, γ-Fe_2_O_3_)/graphene Z-scheme photocatalysts with excellent activity for oxygen evolution under visible light irradiation. RSC Adv..

[30] Nouri L., Hemidouche S., Boudjemaa A., Kaouah F., Sadaoui Z., Bachari K. (2020). Elaboration and characterization of photobiocomposite beads, based on titanium(IV) oxide and sodium alginate biopolymer, for basic blue 41 adsorption/photocatalytic degradation. Int. J. Biol. Macromol..

[31] McIntyre N.S., Zetaruk D.G. (1977). X-ray photoelectron spectroscopic studies of iron oxides. Anal. Chem..

[32] Li Z.-S., Tang X.-K., Liu K., Huang J., Xu Y.-Y., Peng Q., Ao M.L. (2018). Synthesis of a MnO_2_/Fe_3_O_4_/diatomite nanocomposite as an efficient heterogeneous Fenton-like catalyst for methylene blue degradation. Beilstein J. Nanotechnol..

[33] Grosvenor A.P., Kobe B.A., Biesinger M.C., McIntyre N.S. (2004). Investigation of multiplet splitting of Fe 2p XPS spectra and bonding in iron compounds. Surf. Interface Anal..

[34] Biesinger M.C., Payne B.P., Grosvenor A.P., Lau L.W.M., Gerson A.R., Smart R.S.C. (2011). Resolving surface chemical states in XPS analysis of first row transition metals, oxides and hydroxides: Cr, Mn, Fe, Co and Ni. Appl. Surf. Sci..

[35] Fan L.-H., Lu Y.-Q., Yang L.-Y., Huang F.-F., Ouyang X.-K. (2019). Fabrication of polyethylenimine-functionalized sodium alginate/cellulose nanocrystal/polyvinyl alcohol core-shell microspheres ((PVA/SA/CNC)@PEI) for diclofenac sodium adsorption. J. Colloid Interface Sci..

[36] Han Y.-Y., Wu X.-D., Zhang X.-X., Zhou Z.-H., Lu C.-H. (2016). Dual functional biocomposites based on polydopamine modified cellulose nanocrystal for Fe3+-pollutant detecting and autoblocking. ACS Sustain. Chem. Eng..

[37] Chen D.-Y., Wang Y., Liu Y.-X., Cen K.-H., Cao X.-B., Ma Z.-Q., Li Y.-J. (2019). Comparative study on the pyrolysis behaviors of rice straw under different washing pretreatments of water, acid solution, and aqueous phase bio-oil by using TG-FTIR and Py-GC/MS. Fuel.

[38] Pan H., Sun G., Zhao T., Wang G.H. (2015). Thermal properties of epoxy resins crosslinked by an aminated lignin. Polym. Eng. Sci..

[39] Chen D.-Y., Gao A.-J., Ma Z.-Q., Fei D.-Y., Chang Y., Shen C. (2018). In-depth study of rice husk torrefaction: Characterization of solid, liquid and gaseous products, oxygen migration and energy yield. Bioresour. Technol..

[40] Ge Y.-Y., Song Q.-P., Li Z.-L. (2015). A Mannich base biosorbent derived from alkaline lignin for lead removal from aqueous solution. J. Ind. Eng. Chem..

[41] Wang H.-R., Chen X.-J., Zhang L.-D., Li Z.-H., Fan X.-L., Sun S.-L. (2021). Efficient production of lignin-based slow-release nitrogen fertilizer via microwave heating. Ind. Crops Prod..

[42] Ku M.K., Ahn Y., Song Y., Yang Y.H., Kim H. (2014). Effect of oxidized alginate on its electrospinnability. Fibers Polym..

[43] Wang W., Liu M.-Y., Shafiq M., Li H.-Y., Hashim R., El-Newehy M., El-Hamshary H., Morsi Y., Mo X.-M. (2023). Synthesis of oxidized sodium alginate and its electrospun bio-hybrids with zinc oxide nanoparticles to promote wound healing. Int. J. Biol. Macromol..

[44] Li T., Lü S.-Y., Ji Y.-Z., Qi T.-M., Liu M.-Z. (2018). A biodegradable Fe-fertilizer with high mechanical property and sustainable release for potential agriculture and horticulture applications. New J. Chem..

[45] Li Z.-H., Cai M., Yang K., Sun P.-L. (2019). Kinetic study of d-limonene release from finger citron essential oil loaded nanoemulsions during simulated digestion in vitro. J. Funct. Foods.

[46] Salimi M., Motamedi E., Motesharezedeh B., Hosseini H.M., Alikhani H.A. (2020). Starch-g-poly(acrylic acid-co-acrylamide) composites reinforced with natural char nanoparticles toward environmentally benign slow-release urea fertilizers. J. Environ. Chem. Eng..

[47] Arafa E.G., Sabaa M.W., Mohamed R.R., Elzanaty A.M., Abdel-Gawad O.F. (2022). Preparation of biodegradable sodium alginate/carboxymethylchitosan hydrogels for the slow-release of urea fertilizer and their antimicrobial activity. React. Funct. Polym..

[48] Kaur J., Sharma K., Kaushik A. (2023). Waste hemp-stalk derived nutrient encapsulated aerogels for slow release of fertilizers: A step towards sustainable agriculture. J. Environ. Chem. Eng..

[49] Wang W.-S., Yang S.-Q., Zhang A.-P., Yang Z.-L. (2021). Synthesis of a slow-release fertilizer composite derived from waste straw that improves water retention and agricultural yield. Sci. Total Environ..

[50] Kumi A.G., Ibrahim M.G., Nasr M. (2022). Petrochemical wastewater treatment by eggshell modified biochar as adsorbent: A techno-economic and sustainable approach. Adsorpt. Sci. Technol..

[51] Lesbayev B., Auyelkhankyzy M., Ustayeva G., Yeleuov M., Rakhymzhan N., Maltay A., Maral Y. (2023). Recent advances: Biomass-derived porous carbon materials. Sustain. Chem. Environ..

[52] Chen H., Qiu X., Xia T., Li Q., Wen Z., Huang B., Li Y. (2023). Mesoporous materials make hydrogels more powerful in biomedicine. Gels.

[53] Lawrencia D., Wong S.K., Low D.Y., Goh B.H., Goh J.K., Ruktanonchai U.R., Soottitantawat A., Lee L.H., Tang S.Y. (2021). Controlled release fertilizers: A review on coating materials and mechanism of release. Plants.

[54] Tanan W., Panichpakdee J., Suwanakood P., Saengsuwan S. (2021). Biodegradable hydrogels of cassava starch-g-polyacrylic acid/natural rubber/polyvinyl alcohol as environmentally friendly and highly efficient coating material for slow-release urea fertilizers. J. Ind. Eng. Chem..

