# Supplementary material for: Preparation of Biomass-Based Iron-Containing Microspheres from Rice Straw: Enhancing the Water Absorption Performance of Slow-Release Fertilizer
Source: Gels. 2026 Aug 5;12(8):700. doi: 10.3390/gels12080700 (PMC13511771; doi:10.3390/gels12080700)

Supplementary material

Table S1. Results of the central composite experiment for the preparation of iron-containing microspheres.

Run	Mass ratio of OSA to ARS X_1 (g: g)	Ratio of the total mass of the ARS and OSA to the mass of the deionized water X_2 (g: mL)	Volume ratio of the oil phase and aqueous phase X_3 (mL: mL)	Weight concentration of $Fe(NO_3)_3$ solution X_4 (%)	Iron content Y_1 (%)	Water absorption rate Y_2 (%)
1	2:1	1:3	1.5:1	6	6.90	459.07
2	4:1	1:3	1.5:1	6	7.48	499.14
3	2:1	1:5	1.5:1	6	6.94	386.95
4	4:1	1:5	1.5:1	6	7.52	465.94
5	2:1	1:3	1.5:1	8	7.34	420.9
6	4:1	1:3	1.5:1	8	8.57	514.64
7	2:1	1:5	1.5:1	8	7.42	327.79
8	4:1	1:5	1.5:1	8	8.63	453.47
9	2:1	1:3	2.5:1	6	7.42	524.6
10	4:1	1:3	2.5:1	6	8.78	430.93
11	2:1	1:5	2.5:1	6	7.42	489.63
12	4:1	1:5	2.5:1	6	9.29	493.14
13	2:1	1:3	2.5:1	8	7.56	471.89
14	4:1	1:3	2.5:1	8	10.50	441.95
15	2:1	1:5	2.5:1	8	7.60	433.36
16	4:1	1:5	2.5:1	8	10.87	523.76
17	1:1	1:4	2:1	7	6.33	352.9
18	5:1	1:4	2:1	7	10.50	458.09
19	3:1	1:2	2:1	7	7.87	460.84
20	3:1	1:6	2:1	7	7.88	451.18
21	3:1	1:4	2:1	5	7.47	533.24
22	3:1	1:4	2:1	9	8.66	496.74
23	3:1	1:4	1:1	7	7.22	424.04
24	3:1	1:4	3:1	7	9.32	499.18
25	3:1	1:4	2:1	7	7.90	475.13
26	3:1	1:4	2:1	7	8.02	493.12
27	3:1	1:4	2:1	7	8.02	483.55
28	3:1	1:4	2:1	7	8.16	480.41
29	3:1	1:4	2:1	7	8.20	494.81
30	3:1	1:4	2:1	7	8.38	495.24

Table S2. ANOVA analysis results of the quadratic response surface model of iron content and water absorption rate.

Factors	Fe content (%)		Water absorption ratio(%)	
	F-value	P-value	F-value	P-value
Model	47.27	<0.0001	23.03	<0.0001
X_1	380.31	<0.0001	57.06	<0.0001
X_2	1.09	0.3137	9.20	0.0084
X_3	137.17	<0.0001	39.45	<0.0001
X_4	69.40	<0.0001	11.66	0.0038
X_1X_2	0.8473	0.3719	26.41	0.0001
X_1X_3	42.71	<0.0001	43.04	<0.0001
X_1X_4	22.57	0.0003	20.00	0.0004
X_2X_3	0.6037	0.4493	34.61	<0.0001
X_2X_4	0.0029	0.9580	0.3443	0.5661
X_3X_4	0.3287	0.5749	0.2308	0.6378
X_1^2	2.89	0.1099	58.82	<0.0001
X_2^2	2.13	0.1654	8.74	0.0098
X_3^2	0.7299	0.4064	5.92	0.0280
X_4^2	0.1209	0.7329	6.50	0.0222
Lack of Fit	2.17	0.2035	3.57	0.0863

Table S3. The analysis data of release kinetics of iron-containing microspheres in water and soil.

Kinetic models	Equation	Parameter	Water		Soil	
			Fe	N	Fe	N
Zero order	$\frac{M_t}{M_\infty} = k_0 t$	R^2	0.7761	0.9422	0.9852	0.9912
		k_0	0.0271	0.0286	0.0271	0.0261
First order	$\frac{M_t}{M_\infty} = 1 - e^{-k_1 t}$	R^2	0.9893	0.9897	0.8023	0.8744
		k_1	-0.1317	-0.0886	-0.0719	-0.0562
Baker Lonsdale	$\frac{3}{2}\left[1-\left(1-\frac{M_t}{M_\infty}\right)^{\frac{2}{3}}\right] - \frac{M_t}{M_\infty} = K(t)^{\frac{1}{2}}$	R^2	0.9720	0.9622	0.7609	0.7586
		k_B	0.3351	0.3112	0.2568	0.2395
Higuchi	$\frac{M_t}{M_\infty} = k_H t^{1/2}$	R^2	0.9219	0.9963	0.9062	0.9135
		k_H	0.1861	0.1856	0.1874	0.1804
Hixson-Crowell	$(\frac{M_t}{M_\infty})^{1/3} - {M_0}^{1/3} = k_H t$	R^2	0.9600	0.9118	0.8581	0.8229
		k_{HC}	-0.025	-0.0229	-0.0192	-0.0163
Niebergull	$\left(1-\frac{M_t}{M_\infty}\right)^{1/2} = k_N \mathrm{t}$	R^2	0.9381	0.9781	0.9310	0.9192
		K_N	-0.0291	-0.0282	-0.0235	-0.0220

Note: (M_t/M_∞) represents the fractional nutrient release at time t. M_t is the cumulative release rate in time t, M_∞ is the total amount of nutrient element released at the longest time of the experiment. k is a kinetic constant, n is a diffusional exponent characterizing the release mechanism.

Table S4. Korsmeyer–Peppas fitting of the early iron and nitrogen release from iron-containing microspheres.

Medium	Equation	Element	k_{KP}	Fit range	n	R^2
Water	$\frac{M_t}{M_\infty} = k_{kp} t^n$	Fe	0.19876	F≤0.60	0.664	0.9900
		N	0.17106	F≤0.60	0.538	0.9653
Soil		Fe	0.02149	F≤0.60	0.884	0.9239
		N	0.02092	F≤0.60	0.940	0.9383

Note: The model was fitted using the early-release data (F≤0.60), where F denotes the cumulative release fraction, k_{KP} is the kinetic constant, and n is the release exponent. The R^2 values were obtained by linear regression of ln(F) against ln(t). The unit of k_{KP} is d^{-n}. Based on the approximately spherical morphology of the microspheres, n=0.43 and n=0.85 were adopted as the reference values for Fickian diffusion and Case II transport, respectively.

Table S5. EDS analysis data of the iron-containing microspheres.

Distribution diagram Total spectrum diagram			
Element	Line type	Wt%	At%
C	K-series	36.81	47.29
N	K-series	11.85	13.06
O	K-series	36.74	35.44
P	K-series	0.79	0.4
K	K-series	0.01	0.01
Fe	K-series	13.79	3.81
gross:		100.00	100.00

Figure S1. The distribution diagram total spectrum diagram for EDS analysis of the iron-containing microspheres.

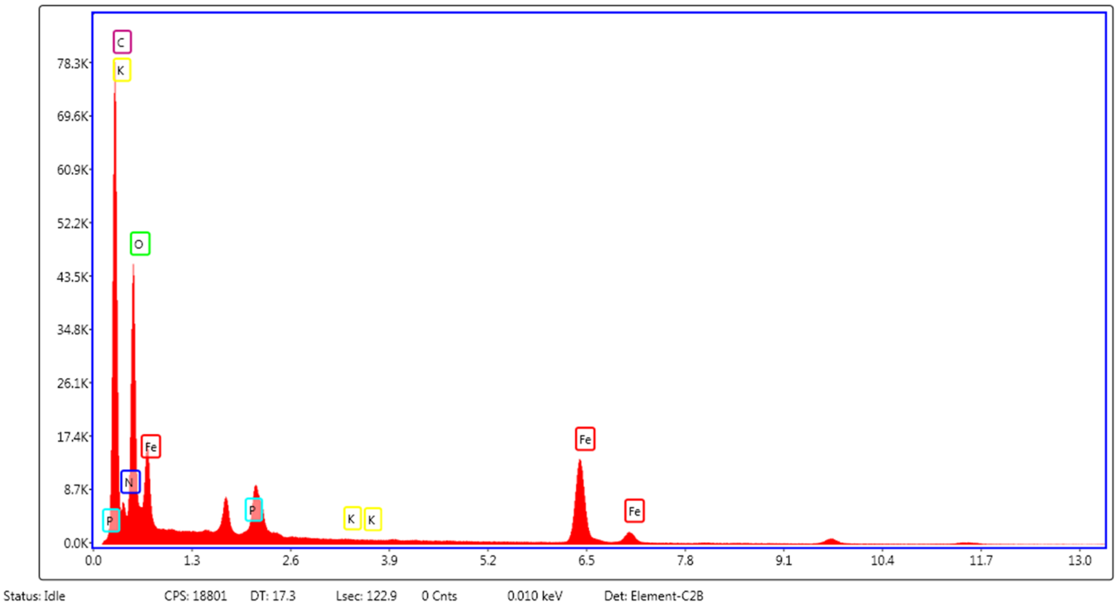

Supplement: Supplementary file 1 [file gels-12-00700-s001.zip › gels-4415535-supplementary.pdf]
